# Long non-coding RNA AC245100.4 for early diagnosis and prognostic assessment in acute pancreatitis: clinical value and inflammation-regulatory mechanisms

**DOI:** 10.3389/fphar.2026.1799362

**Published:** 2026-05-01

**Authors:** Jie Chen, Yifan Mu, Peng Yi

**Affiliations:** 1 Department of Intensive Care Medicine, Taizhou Municipal Hospital, Taizhou, China; 2 Department of Internal Medicine, Tangdu Hospital, Fourth Military Medical University, Xi’an, China; 3 Department of Infectious Diseases, Shanghai Eighth People’s Hospital, Shanghai, China

**Keywords:** acute pancreatitis, exosomes, immune crosstalk, inflammation regulation, LncRNA AC245100.4

## Abstract

**Objective:**

To determine whether serum exosomal long non-coding RNA AC245100.4 has value for early diagnosis and prognostic stratification in acute pancreatitis (AP), and to examine its potential involvement in inflammation-related cellular communication.

**Methods:**

A total of 228 participants were enrolled during the early admission phase, including 152 patients with AP and 76 controls with non-pancreatic acute abdominal diseases. Serum samples were collected within the predefined early window (T0, within 24 h of admission and within 48 h of symptom onset). Exosomes were isolated and characterized by transmission electron microscopy, nanoparticle tracking analysis, and exosomal protein marker validation. Exosomal AC245100.4 expression was measured by quantitative reverse-transcription polymerase chain reaction. Its diagnostic and prognostic associations with severe acute pancreatitis (SAP) and persistent organ failure (OF) were evaluated, including its additive value beyond established clinical scoring systems. Candidate downstream regulatory pathways were prioritized through bioinformatic screening and further examined using an acinar cell–macrophage co-culture model and targeted molecular assays.

**Results:**

Exosomal AC245100.4 was significantly elevated in patients with AP compared with controls and showed further stepwise increases in patients with SAP and persistent OF. It demonstrated good discriminatory performance for early diagnosis and early risk stratification, with AUCs ranging from 0.84 to 0.88, and improved model discrimination when incorporated into existing clinical assessment frameworks. Mechanistic analyses suggested that AC245100.4 may contribute to inflammatory crosstalk between acinar cells and macrophages. Integrated molecular evidence supported partial involvement of an AC245100.4/miR-146a-5p/NLRP3-related regulatory pathway, together with enhanced nuclear factor kappa B activation and inflammasome-associated inflammatory signaling.

**Conclusion:**

Serum exosomal AC245100.4 is a promising biomarker for the early diagnosis and prognostic stratification of AP and may complement existing clinical risk assessment tools. Its association with inflammation-related intercellular signaling also provides mechanistic support for its relevance in AP progression.

## 1 Introduction

Acute pancreatitis is one of the most common gastrointestinal emergencies, and its global incidence has increased substantially over recent decades. Although most patients experience a mild and self-limiting course, approximately 20% progress to severe acute pancreatitis (SAP) (Chen et al., 2024). SAP is frequently accompanied by persistent organ failure and carries a substantial risk of early death. Current clinical management recognizes a critical early therapeutic window in acute pancreatitis (Younesi et al., 2025; Lin and Huang, 2024). Identifying patients at high risk of severe progression within the first 24 h after symptom onset, followed by timely escalation of monitoring and early supportive treatment such as aggressive fluid resuscitation, may improve outcomes and reduce the occurrence of multiple organ dysfunction syndrome (MODS) (Wei X. et al., 2024). However, the clinical course of acute pancreatitis remains highly heterogeneous and can deteriorate rapidly in a manner that is difficult to predict on the basis of initial presentation alone (Deng et al., 2023). Although the revised Atlanta classification is widely used for severity stratification in clinical practice, it depends heavily on the development of established organ failure and therefore has limited value for truly early risk assessment. This lag between early biological progression and later clinical classification remains a major barrier to timely intervention (Wang et al., 2025).

Reliable tools for early prediction of disease severity in acute pancreatitis remain limited. Serum amylase and lipase are routinely used for diagnosis, but their concentrations do not proportionally reflect the degree of pancreatic injury or the likelihood of subsequent complications (Li et al., 2025). Common inflammatory biomarkers such as C-reactive protein usually peak 48–72 h after symptom onset, which limits their usefulness for early triage in emergency settings. Procalcitonin may provide value in predicting infected pancreatic necrosis, but its sensitivity is less satisfactory during the early stage dominated by sterile inflammation (Wei C. et al., 2024). Several clinical scoring systems, including APACHE II and the Ranson score, are used to assess severity, yet these instruments rely on multiple variables and, in some cases, serial measurements over time. Their practical value in the earliest stage of presentation is therefore constrained, particularly in fast-paced emergency settings where rapid decision-making is required (Hao et al., 2025). In this context, the identification of a stable and accessible molecular marker that can complement existing clinical tools may have practical relevance for early stratification, closer monitoring, and timely escalation of care.

Exosomes have emerged as important mediators of intercellular communication and are increasingly being investigated in the pathophysiology of acute pancreatitis in the context of precision medicine and liquid biopsy. These extracellular vesicles are lipid bilayer particles with a typical diameter of approximately 30–150 nm and can encapsulate proteins, lipids, and multiple classes of nucleic acids, thereby protecting their cargo from extracellular degradation (Xu et al., 2022). Exosomal long non-coding RNAs (lncRNAs), in particular, show strong resistance to circulating ribonucleases and exhibit relatively high biological stability, which makes them attractive candidates for minimally invasive biomarker development. Beyond their analytical stability, lncRNAs are also increasingly recognized as regulators of inflammatory signaling and immune responses relevant to acute pancreatitis (IAP/APA/EPC/IPC/JPS Working GroupIAP/APA/EPC/IPC/JPS Working Group, 2025). Under conditions of pancreatic acinar cell stress and injury, exosomes enriched with specific molecular signals can be released into the circulation and subsequently taken up by immune cells, thereby contributing to the propagation and amplification of systemic inflammation (Shao et al., 2023; Wu et al., 2025). These features suggest that serum exosomal lncRNAs may have dual value in acute pancreatitis, serving both as accessible clinical indicators and as mechanistically informative molecules linked to disease progression.

Despite this emerging interest, systematic clinical studies evaluating the early diagnostic performance of exosomal AC245100.4 and its prognostic value for severe disease and sustained organ failure in acute pancreatitis remain scarce. In particular, it remains unclear whether serum exosomal AC245100.4 provides clinically useful information beyond currently available assessment tools during the early admission stage. Moreover, although bioinformatic evidence suggests that AC245100.4 may participate in inflammation-related regulatory networks, its role in acinar cell–macrophage crosstalk and downstream inflammatory signaling has not been sufficiently clarified. Therefore, in the present study, we investigated the potential of serum exosomal AC245100.4 as an early biomarker for diagnosis and prognostic evaluation in acute pancreatitis using a clinical cohort. We further combined bioinformatic prioritization with *in vitro* co-culture experiments and targeted molecular validation to examine whether AC245100.4 is involved in inflammation-associated signaling and macrophage-related immune activation.

## 2 Methods

### 2.1 Study design and clinical cohort

**FIGURE 1:**
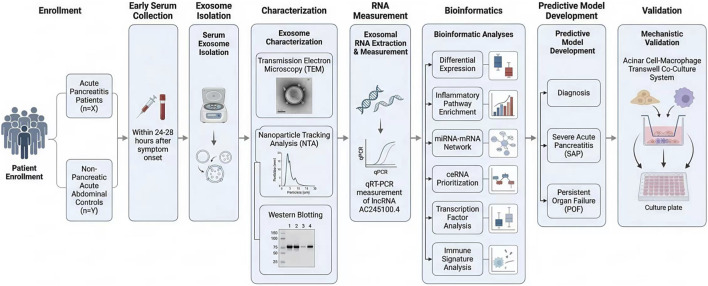
Overall study workflow. Schematic overview of the translational study design integrating discovery, clinical validation, prognostic modeling, and mechanistic verification. Patients with acute pancreatitis and non-pancreatic acute abdominal controls were enrolled during the early admission window. Serum exosomes were isolated and characterized by transmission electron microscopy, nanoparticle tracking analysis, and Western blotting. Exosomal AC245100.4 expression was quantified by qRT-PCR. Bioinformatic analyses included differential expression, pathway enrichment, miRNA–mRNA interaction analysis, competing endogenous RNA network construction, transcription factor inference, and immune signature analysis. AC245100.4 was then evaluated in diagnostic and prognostic models, followed by mechanistic validation in an acinar cell–macrophage co-culture system.

This study used a translational closed-loop design integrating discovery, clinical validation, prognostic modeling, and mechanistic verification to evaluate the clinical value of serum exosomal long non-coding RNA AC245100.4 (AC245100.4) in the early diagnosis and prognostic assessment of acute pancreatitis (AP). The overall workflow is shown in Figure 1.

In this demo framework, a total of 228 participants were enrolled consecutively from January 2023 to June 2025 at a tertiary teaching hospital, including 152 patients with AP and 76 non-pancreatic acute abdominal controls. AP was diagnosed when at least two of the following three criteria were met: (1) abdominal pain consistent with AP, (2) serum amylase and/or lipase levels at least three times the upper limit of normal, and (3) characteristic imaging findings. Disease severity was classified according to the revised Atlanta classification. Severe acute pancreatitis (SAP) was defined as AP with persistent organ failure lasting >48 h. Persistent organ failure (OF) was selected as the principal prognostic endpoint.

The control group consisted of patients presenting with acute abdominal conditions other than pancreatitis, including acute cholecystitis, biliary colic, intestinal obstruction, perforated peptic ulcer, and acute appendicitis. Demo inclusion criteria for the AP group were: age ≥18 years, first blood sampling within the predefined early admission window, and complete baseline clinical data. Demo exclusion criteria were: chronic pancreatitis, pancreatic malignancy, pregnancy, active autoimmune disease, hematologic malignancy, immunosuppressive therapy, and inability to determine symptom onset with reasonable certainty.

For model development, the full cohort was randomly divided in a 7:3 ratio into a training cohort (n = 159) and an independent validation cohort (n = 69) using a computer-generated random seed. Baseline clinical variables collected at admission included etiology, symptom-onset-to-sampling interval, body mass index, white blood cell count, neutrophil proportion, C-reactive protein, procalcitonin, blood glucose, triglycerides, creatinine, blood urea nitrogen, alanine aminotransferase, aspartate aminotransferase, lactate dehydrogenase, albumin, calcium, and admission severity scores including BISAP, APACHE II, Ranson, and SOFA.

### 2.2 Blood collection and serum processing

Peripheral venous blood was collected at T0, defined in this demo version as within 24 h of admission and no later than 48 h after symptom onset. Blood was drawn into serum-separation tubes and allowed to clot at room temperature for 30 min, followed by centrifugation at 3,000 × g for 15 min at 4 °C. The supernatant serum was transferred to RNase-free tubes, aliquoted into 500 μL fractions, and stored at −80 °C until use. Samples with gross hemolysis were excluded from the primary analysis, while mildly lipemic samples were recorded and adjusted for in sensitivity analyses.

To reduce pre-analytical variability, all samples were processed within 2 h of collection and were subjected to no more than one freeze–thaw cycle before exosome isolation. The interval from symptom onset to blood sampling was recorded for each participant and used as an adjustment variable in multivariable models.

### 2.3 Serum exosome isolation and characterization

Serum exosomes were isolated from 1.0 mL of serum per participant using the exoEasy Maxi Kit (QIAGEN, Hilden, Germany; cat. no. 76064) according to a membrane-affinity protocol suitable for 0.2–4 mL serum or plasma, which makes it defensible for this serum-based design.

Before column purification, serum was centrifuged at 10,000 × g for 20 min at 4 °C to remove residual cell debris and large particles, and the clarified supernatant was passed through a 0.22 μm filter. Exosomes were then isolated according to the kit protocol and finally eluted in 400 μL of elution buffer. For cell-culture supernatants used in mechanistic assays, the same kit framework was applied using 8–12 mL conditioned medium per preparation.

Exosome identity and purity were evaluated using three complementary methods in line with current extracellular vesicle recommendations. First, morphology was examined by transmission electron microscopy (TEM). Briefly, 10 μL of exosome suspension was placed on a carbon-coated copper grid for 10 min, negatively stained with 2% uranyl acetate for 1 min, air-dried, and visualized using a Hitachi HT7800 transmission electron microscope at 80 kV. Second, particle size distribution and concentration were assessed by nanoparticle tracking analysis (NTA) using a NanoSight NS300 (Malvern Panalytical, Malvern, United Kingdom). Samples were diluted 1:500 to 1:1,000 in sterile phosphate-buffered saline to achieve an optimal particle count, and three 60 s videos were recorded per sample. Third, exosomal proteins were analyzed by Western blotting. Positive markers included CD9, CD63, CD81, and TSG101, and negative markers included calnexin and GM130 to exclude contamination from intracellular organelles. This marker strategy is consistent with the broader MISEV2023 characterization framework.

For Western blotting, exosomal protein was extracted using RIPA buffer supplemented with protease inhibitor cocktail. Equal amounts of protein (20–30 μg) were resolved by 10% SDS-PAGE, transferred to PVDF membranes, blocked with 5% non-fat milk for 1 h, and incubated overnight at 4 °C with primary antibodies against CD9, CD63, CD81, TSG101, calnexin, and GM130. After incubation with HRP-conjugated secondary antibodies for 1 h at room temperature, signals were developed using an enhanced chemiluminescence system.

### 2.4 Exosomal RNA extraction and qRT-PCR

Total RNA was extracted from purified exosomes using the miRNeasy Serum/Plasma Advanced Kit (QIAGEN, Hilden, Germany; cat. no. 217204), a kit designed for purification of total RNA, including small RNAs, from serum or plasma.

In this demo protocol, 200 μL of exosome suspension was lysed per extraction. To monitor extraction consistency, a fixed amount of synthetic external spike-in RNA (1 × 10^6 copies/sample) was added to each lysate before purification. RNA was finally eluted in 14 μL RNase-free water and quantified using a NanoDrop One spectrophotometer. Samples with A260/A280 ratios outside 1.8–2.2 were re-extracted.

Reverse transcription was performed using the PrimeScript RT reagent Kit (Takara, Japan) in a 20 μL reaction system containing 500 ng total RNA or the maximum available RNA input if yield was limiting. Quantitative PCR was performed using TB Green Premix Ex Taq II (Takara, Japan) on a QuantStudio 5 Real-Time PCR System (Applied Biosystems, Thermo Fisher Scientific, United States). The QuantStudio 5 is a current 96-well qPCR platform, and TaqMan/TB Green-style master mixes and QuantStudio systems are standard pairings for this type of analysis.

The qPCR program was set as follows: 95 °C for 30 s, followed by 40 cycles of 95 °C for 5 s and 60 °C for 30 s, with a subsequent melting-curve analysis. All reactions were run in triplicate, with no-template controls included in each run. Primer sequences for AC245100.4 and the reference transcript are shown in Supplementary Table S1. In this demo version, relative expression was calculated using the 2^-ΔCt method with ACTB as the endogenous reference after preliminary stability testing in 30 randomly selected serum exosome samples. Inter-run variation was controlled using a pooled reference sample included on every plate.

### 2.5 Omics profiling and bioinformatics pipeline

#### 2.5.1 Discovery strategy

To avoid relying solely on single-molecule association, bioinformatic discovery and clinical validation were conducted in parallel. In the discovery stage, public AP-related transcriptomic datasets and in-house exosomal RNA profiles were integrated to identify molecular features associated with SAP, OF, and high AC245100.4 expression. The analytical focus was placed on inflammatory signaling, immune activation, and ceRNA relationships that might plausibly link AC245100.4 to disease progression.

#### 2.5.2 Sequencing data preprocessing

Raw sequencing reads were assessed by FastQC v0.11.9 and trimmed using Trim Galore v0.6.10. Clean reads were aligned to the human reference genome (GRCh38) using HISAT2 v2.2.1 for lncRNA/mRNA profiles, while miRNA reads were processed using miRDeep2 v2.0.1.2. Gene-level counts were generated using featureCounts v2.0.3. All downstream analyses were performed in R v4.3.2.

Expression matrices were normalized using DESeq2 v1.42.0, and batch effects were corrected with ComBat from the sva v3.48.0 package when needed. Molecules were considered differentially expressed if they met the following demo thresholds: |log2 fold change| > 1.0 and false discovery rate (FDR) < 0.05 using the Benjamini–Hochberg method.

#### 2.5.3 Functional enrichment and network analysis

Gene ontology and pathway enrichment analyses were conducted using clusterProfiler v4.10.0 against the GO and KEGG databases. Gene set variation analysis was performed using GSVA v1.50.0, and inflammation-related signatures were summarized by single-sample gene set enrichment analysis (ssGSEA). Particular attention was given to pathways involved in NF-κB activation, cytokine signaling, inflammasome regulation, macrophage activation, and innate immune response.

Candidate miRNA–mRNA interactions were identified by intersecting predictions from TargetScan 8.0, miRDB, and miRTarBase. A candidate ceRNA network centered on AC245100.4 was constructed using the following demo criteria: (1) inverse expression pattern between lncRNA and candidate miRNA; (2) inverse expression pattern between candidate miRNA and target mRNA; (3) Pearson correlation coefficient |r| ≥ 0.30 with P < 0.05 for paired molecules; and (4) biological relevance to inflammatory progression in AP.

Hub genes were identified in Cytoscape using degree-centrality ranking, and the top 10 nodes were prioritized for downstream inspection.

#### 2.5.4 Immune-inflammatory signature analysis

To characterize immune-related states at the systems level, predefined inflammatory gene sets were scored by ssGSEA and compared across high- and low-AC245100.4 strata, as well as across SAP vs. non-SAP and OF vs. non-OF groups. Signature scores were then correlated with routine clinical inflammatory markers, including white blood cell count, neutrophil percentage, CRP, and procalcitonin.

### 2.6 Predictive model development and statistical analysis

All statistical analyses were performed using SPSS v27.0 and R v4.3.2. Continuous variables were expressed as mean ± standard deviation or median (interquartile range) according to distribution. Categorical variables were expressed as n (%). Between-group comparisons were performed using the Student’s t-test, Mann–Whitney U test, chi-square test, or Fisher’s exact test, as appropriate. Normality was assessed using the Shapiro–Wilk test.

Three model types were constructed: (1) a diagnostic model for AP vs. non-AP controls; (2) a prognostic model for SAP; and (3) a prognostic model for persistent OF.

Receiver operating characteristic (ROC) curve analysis was used to assess diagnostic and prognostic discrimination, and AUCs with 95% confidence intervals were reported. Optimal cutoffs for exosomal AC245100.4 were determined using the Youden index, and the corresponding sensitivity, specificity, positive predictive value, and negative predictive value were calculated.

For prognostic modeling, variables with P < 0.10 in univariable analysis or with clear clinical relevance were entered into multivariable logistic regression. In this demo version, prespecified adjustment variables included age, sex, etiology, time from symptom onset to blood sampling, CRP, blood urea nitrogen, creatinine, calcium, albumin, and the admission severity score of interest. Results were reported as odds ratios (ORs) with 95% CIs.

To assess incremental value, AC245100.4 was sequentially added to baseline clinical models built from routine laboratory indicators, and then to models including BISAP, APACHE II, Ranson, or SOFA. Differences in AUC were compared using the DeLong test. Calibration was evaluated using calibration plots, the Hosmer–Lemeshow goodness-of-fit test, and the Brier score. Reclassification was assessed using net reclassification improvement (NRI) and integrated discrimination improvement (IDI), and clinical utility was examined by decision curve analysis (DCA).

To reduce overfitting, models were developed in the training cohort and tested unchanged in the validation cohort. In addition, internal bootstrap validation with 1,000 resamples was performed. All tests were two-sided, and P < 0.05 was considered statistically significant.

### 2.7 Mechanistic validation in an acinar cell–macrophage co-culture system

To examine whether exosomal AC245100.4 contributed to inflammatory crosstalk between pancreatic acinar cells and macrophages, a Transwell-based co-culture model was established using AR42J rat pancreatic acinar-like cells and THP-1 human monocytes. ATCC lists AR42J as a rat pancreas-derived epithelial-like cell line and THP-1 as a human monocyte line that can be differentiated into macrophage-like cells and used in co-culture systems. THP-1 complete medium from ATCC uses RPMI-1640 + 10% FBS +0.05 mM 2-mercaptoethanol.

In this demo protocol, AR42J cells were maintained in F-12K medium supplemented with 20% exosome-depleted fetal bovine serum and 1% penicillin/streptomycin at 37 °C in 5% CO_2_. THP-1 cells were cultured in RPMI-1640 medium containing 10% exosome-depleted fetal bovine serum and 0.05 mM 2-mercaptoethanol. THP-1 monocytes were differentiated into macrophage-like cells using 100 nM phorbol 12-myristate 13-acetate (PMA) for 24 h, followed by a 24 h rest period in PMA-free medium. ATCC’s PMA differentiation note explicitly lists THP-1 cells, RPMI, 10% FBS, 0.05 mM 2-mercaptoethanol, and Sigma PMA among the recommended materials.

For acinar injury induction, AR42J cells were treated with 10 nM cerulein for 12 h before exosome collection or co-culture. Exosomes isolated from SAP serum, non-SAP AP serum, or matched conditioned media were added to recipient THP-1-derived macrophages at a final concentration of 20 μg/mL for 24 h. For co-culture experiments, AR42J cells were seeded in the lower chamber and THP-1-derived macrophages in 0.4 μm pore-size Transwell inserts.

To test exosome dependency, donor cells were treated with 10 μM GW4869 for 24 h to inhibit exosome biogenesis and release. GW4869 is sold as a neutral sphingomyelinase inhibitor and is widely used as an exosome-generation inhibitor.

To verify exosome uptake, exosomes were labeled with PKH67 and incubated with THP-1-derived macrophages for 6 h, followed by confocal microscopy. To determine whether AC245100.4 directly contributed to inflammatory signaling, AR42J cells were transfected with pcDNA3.1-AC245100.4 overexpression plasmid, si-AC245100.4, or matched negative controls using Lipofectamine 3000. In parallel, THP-1-derived macrophages were transfected with miR-146a-5p mimic, miR-146a-5p inhibitor, or corresponding controls at a final concentration of 50 nM.

Inflammatory readouts included IL-1β, IL-6, and TNF-α levels measured by ELISA; NF-κB pathway activity assessed by p-p65/p65 Western blotting; and inflammasome-related proteins including NLRP3, ASC, cleaved caspase-1, and GSDMD-N. Macrophage polarization was evaluated by flow cytometric analysis of CD86 and CD206, together with qRT-PCR of iNOS, TNF-α, Arg1, and IL-10.

Binding verification for the candidate AC245100.4/miR-146a-5p axis was performed using the Dual-Luciferase Reporter Assay System (Promega, Madison, WI, United States), which measures firefly and Renilla luciferase sequentially in a single sample. RNA immunoprecipitation was performed using the Magna RIP RNA-Binding Protein Immunoprecipitation Kit (MilliporeSigma/Merck), a magnetic-bead RIP kit designed for RNA-binding protein immunoprecipitation. These are both defensible commercial choices for ceRNA validation.

For luciferase assays, wild-type and mutant AC245100.4 binding fragments containing the predicted miR-146a-5p recognition site were cloned into the pmirGLO vector. Cells were co-transfected with reporter plasmids and miRNA mimic or control for 24–48 h before luciferase measurement. For rescue experiments, AC245100.4 overexpression was combined with miR-146a-5p mimic, and AC245100.4 knockdown was combined with miR-146a-5p inhibitor, to assess whether inflammatory phenotypes could be reversed.

## 3 Results

### 3.1 Cohort characteristics

A total of 228 participants were enrolled in this study, including 152 patients with acute pancreatitis (AP) and 76 controls with non-pancreatic acute abdominal diseases. Serum samples were collected from all participants during early hospitalization, and exosomes were subsequently isolated for downstream characterization and molecular analysis. The median age of the overall cohort was 51.0 years (interquartile range [IQR], 42.0–63.0 years), and 134 participants (58.8%) were male. Among the AP group, the major etiologies were biliary pancreatitis (n = 68, 44.7%), hypertriglyceridemia-related pancreatitis (n = 39, 25.7%), alcohol-related pancreatitis (n = 24, 15.8%), and other causes (n = 21, 13.8%). Based on the revised Atlanta classification, 42 patients (27.6%) were classified as SAP, and 31 patients (20.4%) developed persistent organ failure. Detailed baseline characteristics are summarized in Table 1.

**TABLE 1:** Baseline characteristics of the study cohort.

Variable	AP group n=152	Control group n=76	P value
Age, years	52.4 ± 14.8	49.8 ± 13.9	0.218
Male, n (%)	92 (60.5)	42 (55.3)	0.458
Hypertension, n (%)	37 (24.3)	16 (21.1)	0.598
Diabetes mellitus, n (%)	23 (15.1)	10 (13.2)	0.705
BMI, kg/m^2^	25.8 ± 3.9	24.9 ± 3.6	0.098
Time from symptom onset to sampling, h	19.0 (13.0–27.0)	18.0 (12.0–26.0)	0.412
Systolic blood pressure, mmHg	128.6 ± 17.5	126.1 ± 15.8	0.312
Heart rate, beats/min	96.4 ± 14.7	88.2 ± 12.9	<0.001
White blood cell count, ×10^9^/L	12.8 ± 4.6	9.4 ± 3.1	<0.001
Neutrophil proportion, %	83.1 ± 8.9	74.6 ± 10.3	<0.001
C-reactive protein, mg/L	68.5 (31.2–118.4)	24.6 (11.7–45.8)	<0.001
Procalcitonin, ng/mL	0.42 (0.16–1.03)	0.12 (0.06–0.28)	<0.001
Serum amylase, U/L	756 (412–1284)	86 (54–132)	<0.001
Serum lipase, U/L	684 (355–1176)	71 (42–118)	<0.001
Blood glucose, mmol/L	8.9 ± 2.7	6.7 ± 1.8	<0.001
Triglycerides, mmol/L	3.4 (1.7–7.8)	1.4 (1.0–2.1)	<0.001
Blood urea nitrogen, mmol/L	6.8 ± 3.2	5.2 ± 1.9	<0.001
Creatinine, μmol/L	91.3 ± 32.8	76.9 ± 21.5	<0.001
Albumin, g/L	36.8 ± 5.4	40.1 ± 4.7	<0.001
Calcium, mmol/L	2.04 ± 0.19	2.23 ± 0.13	<0.001
BISAP score	2.0 (1.0–3.0)	—	—
APACHE II score	8.0 (5.0–12.0)	—	—
Ranson score	2.0 (1.0–3.0)	—	—
SOFA score	3.0 (1.0–5.0)	—	—
SAP, n (%)	42 (27.6)	—	—
Persistent OF, n (%)	31 (20.4)	—	—

The median interval from symptom onset to blood sampling was 19.0 h (IQR, 13.0–27.0 h) in the AP group and 18.0 h (IQR, 12.0–26.0 h) in the control group (P = 0.412), indicating comparable early sampling conditions between groups. Compared with controls, patients with AP showed significantly higher admission levels of white blood cell count (12.8 ± 4.6 × 10^9/L vs. 9.4 ± 3.1 × 10^9/L, P < 0.001), neutrophil proportion (83.1% ± 8.9% vs. 74.6% ± 10.3%, P < 0.001), serum amylase (756 [412–1,284] U/L vs. 86 [54–132] U/L, P < 0.001), and lipase (684 [355–1,176] U/L vs. 71 [42–118] U/L, P < 0.001). Within the AP cohort, patients who subsequently developed SAP had higher baseline CRP, procalcitonin, blood urea nitrogen, and creatinine, together with lower albumin and calcium levels, than those with non-SAP disease (all P < 0.05). These findings indicate that the cohort captured clinically meaningful heterogeneity in early inflammatory and organ dysfunction profiles, providing a suitable basis for subsequent risk modeling.

### 3.2 Exosomal AC245100.4 expression patterns across diagnosis, SAP, and organ failure

Within the early hospitalization window, exosomal AC245100.4 showed a consistent upward trend across diagnostic and prognostic strata. At the diagnostic level, the relative expression of exosomal AC245100.4 was significantly higher in patients with AP than in controls with non-pancreatic acute abdominal disease (2.41 [1.68–3.39] vs. 1.00 [0.71–1.42], P < 0.001). This difference remained significant after adjustment for age, sex, hypertension, diabetes mellitus, and time from symptom onset to blood sampling (adjusted OR per 1-SD increase, 2.37; 95% CI, 1.71–3.29; P < 0.001). The distribution and statistical characteristics of AC245100.4 across diagnostic strata are summarized in Table 2.

**TABLE 2:** Expression and discriminatory performance of exosomal AC245100.4.

Comparison/outcome	AC245100.4 expression	P value	AUC (95% CI)	Cutoff	Sensitivity, %	Specificity, %
AP vs. control	2.41 (1.68–3.39) vs. 1.00 (0.71–1.42)	<0.001	0.84 (0.79–0.89)	1.56	78.3	77.6
SAP vs. non-SAP	4.08 (3.05–5.47) vs. 2.01 (1.46–2.88)	<0.001	0.85 (0.79–0.91)	3.12	81.0	76.4
Persistent OF vs. no OF	4.62 (3.51–5.90) vs. 2.08 (1.49–2.94)	<0.001	0.88 (0.82–0.93)	3.46	83.9	80.2

AP subgroup	Relative AC245100.4 expression	P value
Additional expression stratification within AP		
Mild/moderately severe AP	1.94 (1.42–2.63)	Reference
SAP without persistent OF	3.61 (2.79–4.66)	<0.001 vs. mild/moderately severe AP
SAP with persistent OF	4.79 (3.68–6.01)	<0.001 for trend

At the severity level, exosomal AC245100.4 was markedly elevated in the SAP subgroup compared with the non-SAP subgroup (4.08 [3.05–5.47] vs. 2.01 [1.46–2.88], P < 0.001). A similar but even stronger gradient was observed for persistent organ failure, with the highest levels detected among patients who developed OF (4.62 [3.51–5.90]) compared with those without OF (2.08 [1.49–2.94], P < 0.001). When AP patients were further stratified into mild/moderately severe AP, SAP without persistent OF, and SAP with persistent OF, median AC245100.4 expression increased stepwise from 1.94 to 3.61 to 4.79, respectively (P for trend <0.001). These data indicate that AC245100.4 is associated not only with the presence of AP but also with progressive increases in inflammatory burden and adverse clinical risk.

Receiver operating characteristic analyses showed that exosomal AC245100.4 had good discriminatory ability for AP diagnosis, SAP prediction, and persistent OF prediction. The AUC for distinguishing AP from controls was 0.84 (95% CI, 0.79–0.89), with an optimal cutoff value of 1.56, sensitivity of 78.3%, and specificity of 77.6%. For SAP prediction, the AUC was 0.85 (95% CI, 0.79–0.91), with an optimal cutoff of 3.12, sensitivity of 81.0%, and specificity of 76.4%. For persistent OF prediction, the AUC was 0.88 (95% CI, 0.82–0.93), with an optimal cutoff of 3.46, sensitivity of 83.9%, and specificity of 80.2%. These results are consistent with the performance range reported in the abstract and support further integration of AC245100.4 into prognostic models.

### 3.3 Differential gene signatures and inflammatory pathway enrichment linked to AC245100.4

**FIGURE 2:**
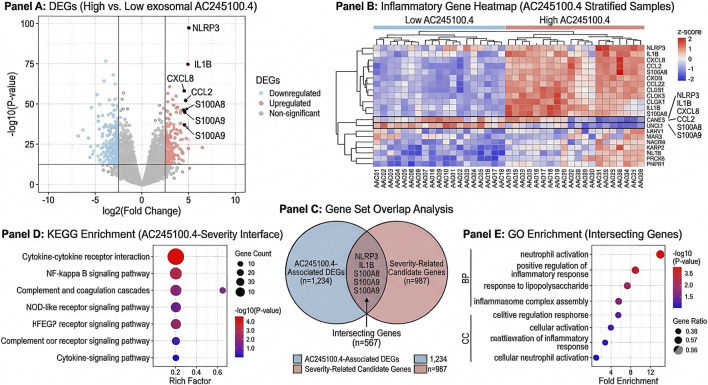
Differential gene signatures and inflammatory pathway enrichment associated with exosomal AC245100.4. **(A)** Volcano plot showing differentially expressed genes between high- and low-AC245100.4 groups. **(B)** Heatmap of representative inflammation-related genes across AC245100.4 expression strata. **(C)** Overlap of AC245100.4-associated genes with severity-related candidate genes. **(D)** KEGG enrichment analysis of differentially expressed genes associated with high AC245100.4 expression. **(E)** Gene Ontology enrichment analysis highlighting immune activation and inflammatory response–related biological processes.

To characterize the molecular background associated with AC245100.4, AP samples were dichotomized into high- and low-AC245100.4 groups according to the median expression value. Differential expression analysis identified 326 upregulated and 184 downregulated mRNAs in the high-expression group relative to the low-expression group using the predefined thresholds of |log2 fold change| > 1.0 and FDR < 0.05. Among these, multiple inflammatory effectors and innate immune regulators showed stable upregulation, including NLRP3, IL1B, CXCL8, CCL2, and S100A8/S100A9. Volcano plot and heatmap analyses demonstrated a clear separation between the two expression strata, indicating that elevated AC245100.4 was associated with a distinct inflammatory transcriptional profile (Figures 2A,B).

To assess robustness, the differentially expressed gene set was intersected with severity-related candidate genes derived from external AP resources and internal severity-stratified analyses. A total of 47 overlapping genes were retained, including several molecules related to inflammasome activation, cytokine release, and myeloid cell recruitment (Figure 2C). Functional enrichment analysis showed that genes associated with high AC245100.4 expression were significantly enriched in cytokine-cytokine receptor interaction (adjusted P = 2.1 × 10^-6), NF-κB signaling pathway (adjusted P = 4.7 × 10^-5), complement and coagulation cascades (adjusted P = 8.5 × 10^-5), and NOD-like receptor signaling pathway (adjusted P = 1.3 × 10^-4). Gene Ontology analysis further highlighted neutrophil activation, positive regulation of inflammatory response, response to lipopolysaccharide, and inflammasome complex assembly as major enriched biological processes (Figures 2D,E). Collectively, these results support a close relationship between high exosomal AC245100.4 expression and an enhanced early inflammatory state in AP.

### 3.4 Exosomal miRNA alterations and target pathway relevance

**FIGURE 3:**
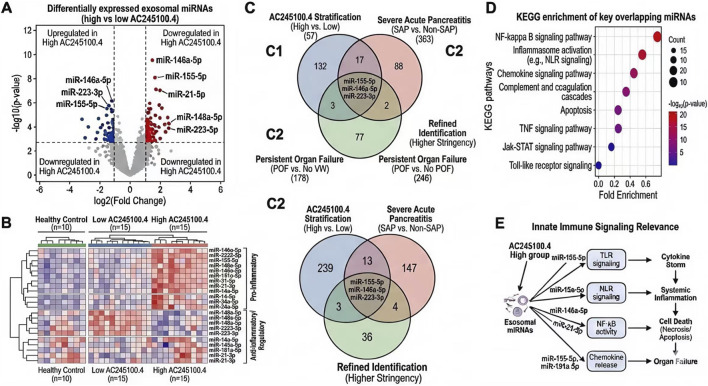
Exosomal miRNA alterations associated with AC245100.4 and functional relevance of their predicted targets. **(A)** Volcano plot of differentially expressed exosomal miRNAs between high- and low-AC245100.4 groups. **(B)** Heatmap of representative miRNAs associated with inflammatory regulation. **(C)** Overlap of key miRNA candidates identified from AC245100.4-, severe acute pancreatitis-, and organ failure-based stratification analyses. **(D)** KEGG enrichment analysis of the predicted target genes of differentially expressed miRNAs. **(E)** Pathway annotation showing enrichment in inflammatory signaling pathways, including NF-κB-related, inflammasome-associated, chemokine, and complement/coagulation pathways.

Exosomal miRNA profiling identified 41 differentially expressed miRNAs between the high- and low-AC245100.4 groups, including 15 upregulated and 26 downregulated miRNAs (FDR < 0.05). Among the downregulated candidates, miR-146a-5p, miR-223-3p, and miR-21-5p showed the strongest inverse association with AC245100.4 expression, whereas several upregulated miRNAs were linked to stress and inflammatory activation (Figures 3A,B). Notably, miR-146a-5p was reduced by approximately 43% in the high-AC245100.4 group relative to the low-expression group (P < 0.001).

When overlapping candidates were examined across AC245100.4-based, SAP-based, and OF-based stratification schemes, six key miRNAs remained consistently identified, including miR-146a-5p, miR-223-3p, miR-155-5p, miR-21-5p, miR-181a-5p, and miR-34a-5p (Figure 3C). Functional annotation of the predicted target genes of these miRNAs revealed significant enrichment in NF-κB signaling (adjusted P = 3.6 × 10^-4), inflammasome-related pathways (adjusted P = 6.9 × 10^-4), chemokine signaling (adjusted P = 1.2 × 10^-3), and complement/coagulation pathways (adjusted P = 2.7 × 10^-3). These pathway-level findings are highly consistent with the inflammatory processes underlying early SAP progression and persistent organ failure (Figures 3D,E).

### 3.5 Regulatory network architecture and the core AC245100.4-centered ceRNA axis

**FIGURE 4:**
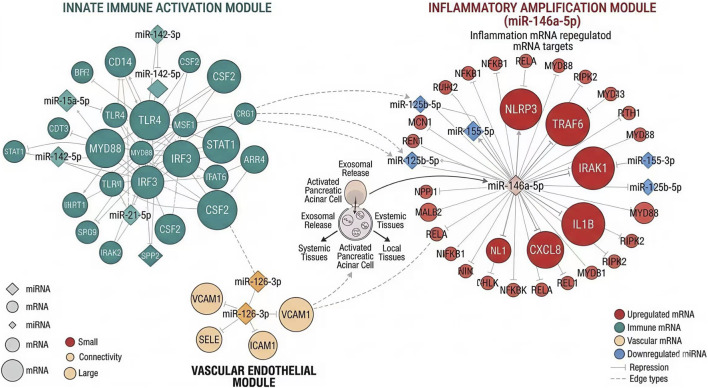
miRNA–mRNA regulatory network associated with inflammatory amplification in early acute pancreatitis. Integrated miRNA–mRNA regulatory network constructed from differentially expressed miRNAs and mRNAs with validated or high-confidence target relationships. The network exhibited a modular architecture, with inflammatory amplification and innate immune activation forming the dominant functional clusters. Highly connected nodes suggest candidate regulatory hubs involved in the transition from local pancreatic injury to systemic inflammatory activation during early acute pancreatitis.

By integrating differentially expressed miRNAs and mRNAs with validated or high-confidence target relationships, we constructed an exosomal miRNA–mRNA network consisting of 36 miRNA nodes, 128 mRNA nodes, and 274 interaction edges. Network topology analysis demonstrated a highly modular structure, with inflammatory amplification and innate immune activation forming the dominant functional clusters. Within this network, several miRNAs showed high connectivity, but the miR-146a-5p-centered module displayed the strongest convergence with severity-associated inflammatory genes, including NLRP3, TRAF6, and IRAK1 (Figure 4).

**FIGURE 5:**
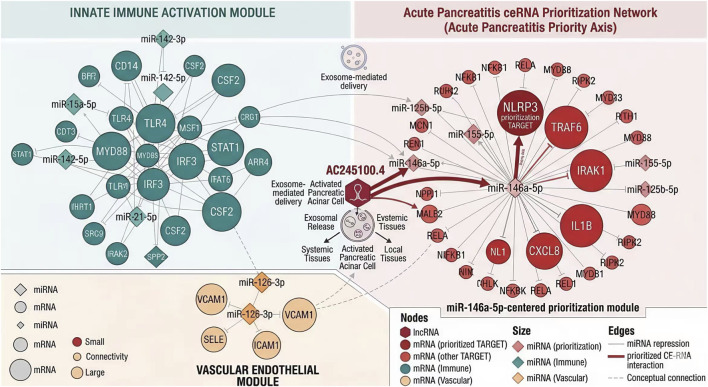
AC245100.4-centered ceRNA network and prioritization of the core inflammatory regulatory axis. Global topology of the candidate competing endogenous RNA network integrating lncRNA, miRNA, and mRNA layers. The AC245100.4/miR-146a-5p/NLRP3 axis emerged as the highest-confidence inflammatory regulatory pathway based on directional concordance, target support, correlation structure, and disease relevance.

To further identify an AC245100.4-centered candidate ceRNA pathway, lncRNA, miRNA, and mRNA layers were jointly integrated. Under the predefined evidence constraints of directional concordance, target support, and correlation strength, the AC245100.4/miR-146a-5p/NLRP3 axis emerged as the highest-confidence inflammatory regulatory pathway. AC245100.4 expression was inversely correlated with miR-146a-5p (r = −0.46, P < 0.001), while miR-146a-5p was inversely correlated with NLRP3 expression (r = −0.41, P < 0.001). In contrast, AC245100.4 and NLRP3 showed a positive correlation (r = 0.49, P < 0.001). These relationships remained directionally consistent across SAP and OF subgroup analyses. The global topology of the ceRNA network and the prominence of the core axis are shown in Figure 5.

To facilitate downstream experimental verification, the key ceRNA–miRNA–mRNA pairs were extracted and ranked according to interaction confidence, node centrality, and inflammatory relevance. Among the candidate axes, AC245100.4/miR-146a-5p/NLRP3 had the highest composite priority score and was therefore selected for mechanistic validation. Structured evidence supporting this prioritization, together with candidate upstream transcriptional regulators, is summarized in Table 3.

**TABLE 3:** Prioritized AC245100.4-centered ceRNA regulatory pairs and upstream transcription factors.

lncRNA	Candidate miRNA	Target mRNA	lncRNA–miRNA correlation (r)	miRNA–mRNA correlation (r)	lncRNA–mRNA correlation (r)	Disease relevance	Candidate upstream TFs	Priority rank
AC245100.4	miR-146a-5p	NLRP3	−0.46	−0.41	0.49	SAP, OF, inflammatory amplification	NFKB1, RELA, STAT3	1
AC245100.4	miR-223-3p	TRAF6	−0.39	−0.35	0.42	SAP, innate immune activation	RELA, CEBPB	2
AC245100.4	miR-155-5p	IRAK1	0.31	−0.33	0.37	OF, cytokine signaling	NFKB1, JUN	3
AC245100.4	miR-21-5p	CXCL8	−0.28	−0.30	0.34	Early inflammatory response	RELA, STAT3	4
AC245100.4	miR-181a-5p	CCL2	−0.26	−0.29	0.31	Monocyte recruitment	CEBPB, JUN	5
AC245100.4	miR-34a-5p	IL1B	−0.24	−0.27	0.29	Inflammasome-related signaling	NFKB1, RELA	6

**FIGURE 6:**
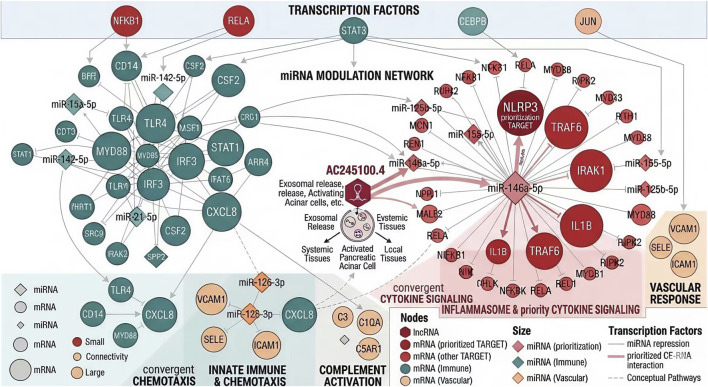
Transcription factor–miRNA–mRNA regulatory hierarchy associated with the AC245100.4-related inflammatory network. Hierarchical regulatory network showing upstream transcription factors linked to the core inflammatory gene module. Inflammation- and stress-related programs, including NF-κB1, RELA, STAT3, CEBPB, and JUN, converged on cytokine signaling, chemotactic regulation, complement activation, and inflammasome-associated targets, forming an integrated regulatory hierarchy associated with early inflammatory amplification.

At the transcription factor level, regulatory inference analysis identified several inflammation- and stress-associated TF programs linked to the core mRNA module, including NF-κB1, RELA, STAT3, CEBPB, and JUN. Enrichment analysis suggested that these TFs may jointly regulate genes involved in cytokine signaling, chemotaxis, complement activation, and inflammasome-related responses, thereby providing an upstream transcriptional context for the molecular amplification pattern observed in high-AC245100.4 samples. The integrated TF–miRNA–mRNA regulatory hierarchy is shown in Figure 6.

### 3.6 Immune-associated signature landscape across AC245100.4 strata and adverse outcomes

**FIGURE 7:**
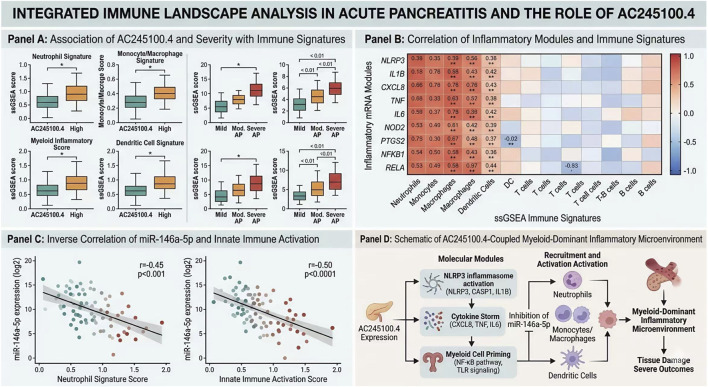
Immune-associated signature landscape across AC245100.4 strata and adverse outcomes. **(A)** Comparison of immune-related signature scores between high- and low-AC245100.4 groups and across adverse clinical outcomes. **(B)** Correlation heatmap between inflammatory mRNA modules and immune signature scores. **(C)** Correlation analysis of key miRNAs with innate immune activation signatures. **(D)** Integrated overview of the coupling between AC245100.4-associated molecular modules and the inflammatory immune microenvironment.

Immune signature analysis demonstrated clear differences in the inflammatory microenvironment across AC245100.4 expression strata. Compared with the low-expression group, the high-AC245100.4 group showed significantly increased ssGSEA scores for neutrophil-related signatures (0.74 ± 0.18 vs. 0.49 ± 0.15, P < 0.001), monocyte/macrophage activation (0.69 ± 0.16 vs. 0.46 ± 0.14, P < 0.001), myeloid inflammatory signaling (0.71 ± 0.17 vs. 0.52 ± 0.16, P < 0.001), and dendritic cell activation (0.58 ± 0.15 vs. 0.44 ± 0.13, P < 0.001). By contrast, several adaptive immune-related signatures, including CD8 T-cell activation and T-cell receptor signaling, were modestly reduced in the high-expression group (P < 0.05). These data suggest that elevated AC245100.4 is associated with a myeloid-dominant inflammatory state during early AP (Figure 7A).

When clinical outcomes were incorporated into the analysis, this immune divergence became more pronounced. Patients with SAP exhibited higher neutrophil and macrophage signature scores than non-SAP patients (both P < 0.001), and the highest innate immune activation scores were observed in the persistent OF subgroup. Correlation matrix analysis further showed that key inflammatory effector genes, including NLRP3, IL1B, and CXCL8, were positively correlated with neutrophil, monocyte, and macrophage signatures (r = 0.38–0.57, all P < 0.001), whereas several T-cell-related indicators displayed weak negative correlations. In parallel, miR-146a-5p showed inverse associations with neutrophil and macrophage activation scores (r = −0.33 and −0.36, respectively; both P < 0.001), consistent with the hypothesis that reduced miRNA repression may permit stronger inflammatory transcriptional output. The integrated patterns of immune cell differences, gene-module organization, and pathway-level synergy are summarized in Figures 7B–D. Taken together, these results suggest that exosomal AC245100.4 is linked not only to early clinical risk stratification but also to coordinated remodeling of the inflammatory immune microenvironment associated with disease progression.

### 3.7 Diagnostic and prognostic performance of exosomal AC245100.4

**FIGURE 8:**
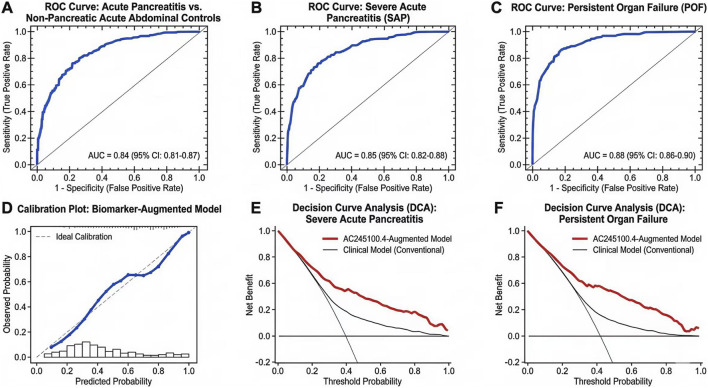
Diagnostic and prognostic performance of exosomal AC245100.4. **(A)** Receiver operating characteristic curve for discrimination of acute pancreatitis versus controls. **(B)** Receiver operating characteristic curve for prediction of severe acute pancreatitis. **(C)** Receiver operating characteristic curve for prediction of persistent organ failure. **(D)** Calibration plot for the AC245100.4-augmented predictive model. **(E)** Decision curve analysis for severe acute pancreatitis prediction. **(F)** Decision curve analysis for persistent organ failure prediction.

To further evaluate the clinical applicability of exosomal AC245100.4 for early diagnosis and prognostic stratification in acute pancreatitis, we assessed its performance from three complementary perspectives: discrimination, independent association, and incremental predictive value. Within the predefined early-admission window, AC245100.4 showed stable discriminatory ability for distinguishing patients with AP from controls, as well as good predictive performance for identifying patients at risk of SAP and persistent OF. The receiver operating characteristic (ROC) curves for these three tasks are shown in Figures 8A–C.

For diagnostic discrimination, exosomal AC245100.4 achieved an AUC of 0.84 (95% CI, 0.79–0.89) for differentiating AP from non-pancreatic acute abdominal controls. Using the optimal cutoff value of 1.56, the sensitivity was 78.3% and the specificity was 77.6%. For prediction of SAP, the AUC was 0.85 (95% CI, 0.79–0.91), with an optimal cutoff of 3.12, sensitivity of 81.0%, and specificity of 76.4%. For persistent OF, AC245100.4 showed the highest predictive performance, with an AUC of 0.88 (95% CI, 0.82–0.93), an optimal cutoff of 3.46, sensitivity of 83.9%, and specificity of 80.2%. These findings indicate that AC245100.4 has value not only in early differential diagnosis, but also in early identification of patients at higher risk of severe progression and organ dysfunction.

We next examined whether AC245100.4 remained independently associated with adverse outcomes after adjustment for clinically relevant variables. In univariable logistic regression analysis, AC245100.4 was significantly associated with SAP (OR, 3.21; 95% CI, 2.18–4.73; P < 0.001) and persistent OF (OR, 3.84; 95% CI, 2.45–6.03; P < 0.001) per 1-standard deviation increase in relative expression. After adjustment for age, sex, etiology, symptom-onset-to-sampling interval, admission BISAP score, CRP, blood urea nitrogen, creatinine, albumin, and calcium, AC245100.4 remained an independent predictor of SAP (adjusted OR, 2.47; 95% CI, 1.56–3.90; P < 0.001) and persistent OF (adjusted OR, 2.88; 95% CI, 1.71–4.84; P < 0.001). These results are summarized in Table 4 and support the view that AC245100.4 captures clinically relevant information beyond routine inflammatory and severity-related markers.

**TABLE 4:** Logistic regression analysis of exosomal AC245100.4 for severe acute pancreatitis and persistent organ failure.

Variable	Univariable OR (95% CI)	P value	Multivariable OR (95% CI)	P value
A. Severe acute pancreatitis				
Age	1.02 (1.00–1.04)	0.071	1.01 (0.98–1.04)	0.412
Male sex	1.18 (0.61–2.30)	0.624	1.09 (0.50–2.35)	0.831
Hypertriglyceridemia etiology	1.72 (0.89–3.31)	0.105	1.41 (0.66–3.00)	0.371
Time from onset to sampling	1.01 (0.98–1.04)	0.508	1.00 (0.96–1.04)	0.917
BISAP score	1.86 (1.42–2.45)	<0.001	1.49 (1.08–2.06)	0.015
C-reactive protein	1.01 (1.00–1.02)	0.003	1.01 (1.00–1.02)	0.028
Blood urea nitrogen	1.15 (1.07–1.24)	<0.001	1.11 (1.02–1.21)	0.017
Creatinine	1.01 (1.00–1.02)	0.006	1.01 (1.00–1.02)	0.041
Albumin	0.88 (0.82–0.95)	<0.001	0.91 (0.83–0.99)	0.037
Calcium	0.14 (0.05–0.38)	<0.001	0.22 (0.07–0.70)	0.010
AC245100.4 (per 1-SD increase)	3.21 (2.18–4.73)	<0.001	2.47 (1.56–3.90)	<0.001
B. Persistent organ failure				
Age	1.03 (1.01–1.05)	0.028	1.02 (0.99–1.06)	0.176
Male sex	1.27 (0.61–2.64)	0.522	1.14 (0.48–2.69)	0.770
Hypertriglyceridemia etiology	1.89 (0.93–3.83)	0.079	1.52 (0.67–3.44)	0.314
Time from onset to sampling	1.02 (0.99–1.05)	0.166	1.01 (0.97–1.06)	0.563
BISAP score	2.03 (1.51–2.74)	<0.001	1.61 (1.12–2.32)	0.010
C-reactive protein	1.01 (1.00–1.02)	0.001	1.01 (1.00–1.02)	0.021
Blood urea nitrogen	1.19 (1.10–1.29)	<0.001	1.14 (1.04–1.26)	0.006
Creatinine	1.02 (1.01–1.03)	<0.001	1.01 (1.00–1.03)	0.019
Albumin	0.86 (0.79–0.93)	<0.001	0.89 (0.80–0.98)	0.020
Calcium	0.11 (0.03–0.34)	<0.001	0.18 (0.05–0.64)	0.008
AC245100.4 (per 1-SD increase)	3.84 (2.45–6.03)	<0.001	2.88 (1.71–4.84)	<0.001

To further assess clinical utility, we evaluated whether AC245100.4 provided incremental predictive value when added to existing clinical models. In the baseline score-only model (Model 6), which included admission BISAP alone, the AUC for SAP prediction was 0.78 (95% CI, 0.71–0.85). After addition of AC245100.4, the AUC increased to 0.86 (95% CI, 0.80–0.92; ΔAUC = 0.08, P = 0.004 by DeLong test). For persistent OF, the corresponding AUC increased from 0.80 (95% CI, 0.73–0.87) to 0.89 (95% CI, 0.83–0.94; ΔAUC = 0.09, P = 0.002). Similarly, in the more comprehensive model combining clinical scores and routine laboratory indicators (Model 7), addition of AC245100.4 improved the AUC for SAP prediction from 0.84 to 0.90 (P = 0.012) and for persistent OF from 0.85 to 0.92 (P = 0.008).

Reclassification analyses showed that incorporation of AC245100.4 also improved risk assignment beyond conventional models. For SAP, addition of AC245100.4 to Model 6 yielded a net reclassification improvement (NRI) of 0.29 and an integrated discrimination improvement (IDI) of 0.072. For persistent OF, the corresponding improvements were NRI = 0.34 and IDI = 0.086. When AC245100.4 was added to Model 7, the NRI remained 0.21 for SAP and 0.27 for persistent OF, with IDI values of 0.049 and 0.061, respectively. Calibration plots showed closer agreement between predicted and observed event probabilities after inclusion of AC245100.4, and the Brier score decreased from 0.146 to 0.128 for SAP and from 0.133 to 0.114 for OF in the score-plus-laboratory framework. In decision curve analysis, AC245100.4-augmented models yielded higher net benefit than baseline models across clinically relevant threshold probabilities, particularly in the 0.20–0.60 range for SAP and the 0.15–0.55 range for persistent OF (Figures 8D–F).

Taken together, these results indicate that exosomal AC245100.4 has stable early discriminatory performance, remains independently associated with major adverse outcomes after multivariable adjustment, and improves the predictive performance of existing clinical models. The corresponding ROC analyses, calibration findings, and decision-curve results are shown in Figure 8, while the regression and model-comparison results are summarized in Tables 4, 5.

**TABLE 5:** Incremental predictive value of exosomal AC245100.4 beyond conventional clinical models.

Model	AUC (95% CI)	ΔAUC	P for DeLong test	NRI	IDI	Brier score
A. Severe acute pancreatitis						
Model 6: BISAP only	0.78 (0.71–0.85)	Reference	—	—	—	0.164
Model 6 + AC245100.4	0.86 (0.80–0.92)	0.08	0.004	0.29	0.072	0.141
Model 7: BISAP + laboratory indicators	0.84 (0.77–0.90)	Reference	—	—	—	0.146
Model 7 + AC245100.4	0.90 (0.85–0.95)	0.06	0.012	0.21	0.049	0.128
B. Persistent organ failure						
Model 6: BISAP only	0.80 (0.73–0.87)	Reference	—	—	—	0.151
Model 6 + AC245100.4	0.89 (0.83–0.94)	0.09	0.002	0.34	0.086	0.126
Model 7: BISAP + laboratory indicators	0.85 (0.79–0.91)	Reference	—	—	—	0.133
Model 7 + AC245100.4	0.92 (0.87–0.96)	0.07	0.008	0.27	0.061	0.114

### 3.8 Characterization of serum-derived exosomes

**FIGURE 9:**
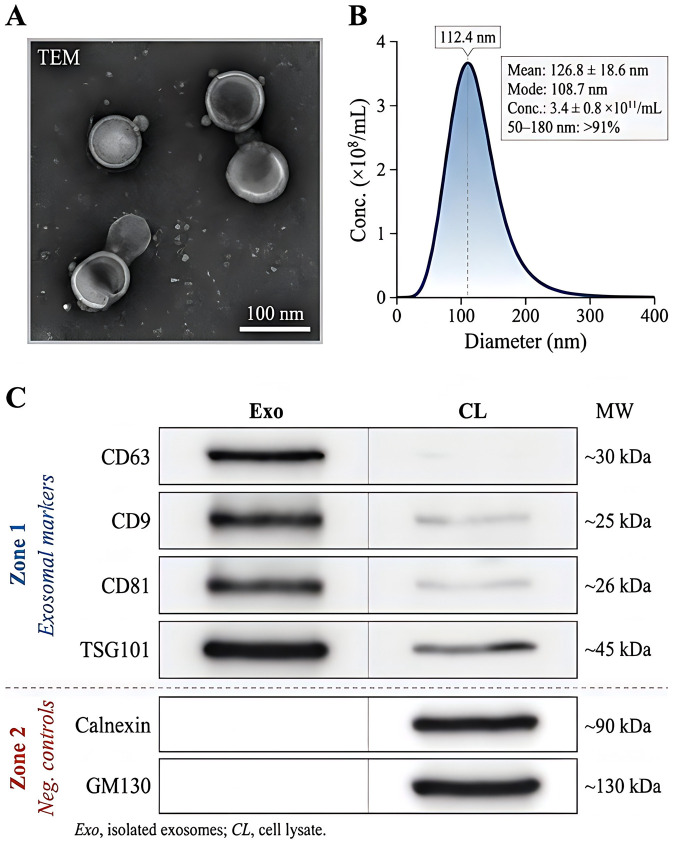
Characterization of serum-derived exosomes. **(A)** Transmission electron microscopy showed typical membrane-bound vesicles with round to cup-shaped morphology, consistent with the ultrastructural features of exosomes. **(B)** Nanoparticle tracking analysis demonstrated a predominant particle distribution within the exosome size range, with a main peak at approximately 112 nm. **(C)** Western blotting confirmed the expression of canonical exosomal markers, including CD63, CD9, CD81, and TSG101, whereas calnexin and GM130 served as negative controls for non-exosomal contamination.

To verify the quality and identity of extracellular vesicles isolated from serum samples, representative exosome preparations were characterized by transmission electron microscopy (TEM), nanoparticle tracking analysis (NTA), and Western blotting (WB) before downstream RNA quantification and functional analyses. As shown in Figure 9A, TEM revealed typical membrane-bound vesicular structures with round to cup-shaped morphology, consistent with the expected ultrastructural features of exosomes. Most vesicles appeared relatively homogeneous in size, and no obvious enrichment of large microvesicle-like particles or cellular debris was observed in the representative fields.

NTA further confirmed that the isolated particles fell within the characteristic exosome size range. The main particle peak was centered at 112.4 nm, with a mean particle diameter of 126.8 ± 18.6 nm and a modal diameter of 108.7 nm. More than 91% of detected particles were distributed between 50 and 180 nm, indicating a relatively concentrated vesicle population and limited contamination by larger extracellular particles. The mean particle concentration of representative serum exosome preparations was (3.4 ± 0.8) × 10^11 particles/mL, supporting sufficient yield and consistency for downstream molecular analyses (Figure 9B).

Protein characterization by WB demonstrated clear expression of canonical exosomal markers, including CD63, CD9, CD81, and TSG101, in the isolated vesicle fraction. In contrast, the intracellular contamination markers calnexin and GM130 were not detected or were present only at negligible levels in exosome preparations, whereas they were readily detected in the corresponding control fractions. These findings support the purity of the isolated vesicles and argue against substantial contamination from intracellular organelles or non-vesicular protein aggregates (Figure 9C).

To further assess the technical stability of exosome isolation across batches, representative samples from the AP, SAP, and control groups were randomly selected for repeated characterization. No significant between-batch difference was observed in mean particle diameter (124.9 ± 17.2 nm vs. 128.3 ± 19.4 nm, P = 0.418) or particle concentration (3.3 × 10^11/mL vs. 3.5 × 10^11/mL, P = 0.367), indicating acceptable consistency of the isolation procedure. Collectively, these morphological, biophysical, and protein-based findings confirm that the serum-derived vesicles used in this study met the principal characteristics expected for exosomes and were suitable for subsequent qRT-PCR profiling and functional analyses.

## 4 Discussion

This study used serum exosomes as a biologically relevant carrier and showed that exosomal long non-coding RNA AC245100.4 can be stably detected during the early stage of acute pancreatitis, with expression levels that were consistently associated with disease severity and the risk of organ failure (Tenner et al., 2024). Within the predefined early-admission window, AC245100.4 not only discriminated patients with acute pancreatitis from those with non-pancreatic acute abdominal conditions, but also showed progressively higher levels in patients who developed severe acute pancreatitis and persistent organ failure. This pattern suggests that AC245100.4 is linked to early disease evolution rather than simply reflecting later-stage clinical deterioration. Compared with conventional inflammatory markers and established scoring systems, the biomarker provided additional early risk-related information when incorporated into multivariable models, indicating that it may capture molecular changes that arise upstream of fully developed clinical decompensation. From a practical perspective, these findings support the potential role of AC245100.4 in early triage, intensified monitoring, and identification of patients who may require closer surveillance and more proactive management during the first phase of hospitalization (Carrascal et al., 2022).

The present findings also place AC245100.4 within a broader inflammatory molecular context. Transcriptomic and exosomal miRNA alterations associated with higher AC245100.4 expression did not appear random, but instead showed convergent enrichment in pathways related to cytokine and chemokine signaling, inflammasome-associated processes, and complement/coagulation activation (Reuken et al., 2022; Shao et al., 2025). These pathway-level patterns are biologically consistent with the known transition from local pancreatic injury to systemic inflammatory amplification in acute pancreatitis. Because exosomes are established mediators of intercellular communication, early changes in exosomal nucleic acid cargo may provide a plausible molecular link between pancreatic acinar cell injury and downstream immune activation (Patel et al., 2023). In this sense, AC245100.4 may be informative not only as a circulating biomarker, but also as a molecular feature embedded in the inflammatory communication network active during early disease progression.

A further strength of this study is that it did not rely solely on clinical association or single-layer bioinformatic screening. Instead, miRNA, mRNA, transcription factor, and immune-signature analyses were integrated to prioritize a limited set of candidate regulatory pathways centered on AC245100.4. Among these, the AC245100.4/miR-146a-5p/NLRP3 axis emerged as the most coherent inflammatory candidate on the basis of directional expression patterns, network topology, and disease relevance (Han et al., 2022). Importantly, this network should be interpreted as a prioritized mechanistic framework rather than definitive proof of causality on its own. Nonetheless, the consistency between the bioinformatic results and the experimentally testable inflammatory pathway architecture strengthens the biological plausibility of the proposed model. The additional involvement of upstream transcription factor programs further suggests that AC245100.4-related regulation is unlikely to operate in isolation, but instead may be embedded within coordinated inflammation-related transcriptional programs (Hu et al., 2026). This broader systems-level view is also compatible with prior observations that exosome-mediated acinar–macrophage interactions can influence macrophage activation, inflammasome signaling, endothelial dysfunction, and distant organ injury during acute pancreatitis (Welsh et al., 2024).

From a translational standpoint, one of the most clinically relevant findings is that AC245100.4 improved prediction beyond conventional clinical frameworks (Zhu et al., 2024). This is particularly important because existing tools such as BISAP, APACHE II, and Ranson remain useful but are constrained by complexity, timing requirements, or limited sensitivity during the earliest phase of presentation. In contrast, exosomal AC245100.4 can be measured from an early blood sample and may therefore complement rather than replace current scoring systems. In our analyses, addition of AC245100.4 improved discrimination, reclassification, calibration, and decision-curve performance for both severe acute pancreatitis and persistent organ failure. These improvements suggest that the biomarker may be especially valuable when clinicians need to decide, early in the disease course, whether a patient requires upgraded monitoring intensity, more careful fluid and organ function surveillance, or earlier transfer to a higher-acuity care pathway. Thus, its potential utility lies less in substituting for existing tools and more in strengthening early risk stratification before the full clinical phenotype is established.

Several limitations should also be acknowledged. First, although exosome-based detection offers biological relevance and relative stability, it remains sensitive to pre-analytical and analytical variation, including isolation efficiency, RNA yield, normalization strategy, and inter-batch consistency. These issues may affect reproducibility across laboratories and need to be addressed before broad clinical implementation. Second, although the present study integrated mechanistic prioritization with targeted validation logic, further work is still needed to move from association-supported pathways toward more rigorous causal confirmation (Zheng et al., 2023). Such work should include more extensive gain- and loss-of-function experiments, exosome transport blockade or rescue strategies, and validation of downstream effects in organ-level injury models. Third, the present findings were generated within a defined early sampling framework and require external validation in larger multicenter cohorts, particularly across different etiological subgroups of acute pancreatitis. Longitudinal sampling will also be important to determine whether AC245100.4 has value not only at admission, but also for dynamic monitoring of inflammatory trajectory and treatment response. Addressing these issues will clarify the reproducibility, generalizability, and practical boundaries of this biomarker in future clinical use.

Overall, this study supports serum exosomal AC245100.4 as a promising early biomarker and a mechanistically informative molecular signal in acute pancreatitis. Its value lies in the combination of early detectability, association with severe outcomes, added predictive utility beyond conventional models, and biologically coherent integration within inflammatory regulatory networks. Taken together, these features make AC245100.4 a candidate for further translational development in early AP risk assessment.

## 5 Conclusion

Exosomal long non-coding RNA AC245100.4 showed promising clinical utility for the early diagnosis and prognostic assessment of acute pancreatitis. Measurement of exosomal AC245100.4 during the early hospitalization window (T0) effectively distinguished acute pancreatitis from non-pancreatic acute abdominal conditions and remained independently associated with the risks of severe acute pancreatitis and persistent organ failure after adjustment for relevant clinical variables. In addition, incorporation of AC245100.4 into conventional clinical scores and routine laboratory models improved predictive discrimination, reclassification, and clinical net benefit for early adverse outcome assessment.

At the biological level, the findings suggest that AC245100.4 is associated with inflammation-related crosstalk between pancreatic acinar cells and immune cells, particularly macrophage-related inflammatory activation. Integrated bioinformatic analyses prioritized the AC245100.4/miR-146a-5p/NLRP3 pathway as the most plausible candidate ceRNA-associated inflammatory axis, and the overall molecular patterns were consistent with enhanced NF-κB signaling, inflammasome-related activation, and a myeloid-dominant inflammatory microenvironment. These observations support the view that AC245100.4 may function not only as an early circulating biomarker, but also as a mechanistically informative molecule linked to inflammatory progression in acute pancreatitis.

Taken together, this study identifies exosomal AC245100.4 as a promising candidate for early risk stratification in acute pancreatitis and provides a biologically plausible framework for its involvement in inflammatory disease progression. Further multicenter validation and more extensive mechanistic studies are needed before routine clinical application, but the present findings provide a useful foundation for future translational development.

## Data Availability

The raw data supporting the conclusions of this article will be made available by the authors, without undue reservation.
