# Supplementary material for: Long non-coding RNA AC245100.4 for early diagnosis and prognostic assessment in acute pancreatitis: clinical value and inflammation-regulatory mechanisms
Source: Front Pharmacol. 2026 May 1;17:1799362. doi: 10.3389/fphar.2026.1799362 (PMC13176236; doi:10.3389/fphar.2026.1799362)
Supplement: Supplementary file 1 [file Table1.docx]

Supplementary Table S1. Primer sequences used for qRT-PCR analysis

| Target gene/transcript | Forward primer  (5′–3′) | Reverse primer  (5′–3′) | Product size  (bp) | Application |
| --- | --- | --- | --- | --- |
| AC245100.4 | AGCTTCTGAGAAGGCTGTGA | TCCACAGGTTCTTCTGGAGT | 142 | qRT-PCR |
| ACTB | CCAACCGCGAGAAGATGA | CCAGAGGCGTACAGGGATAG | 138 | Internal reference for qRT-PCR |
